# Impact of mother’s childhood trauma on development of psychopathological dimensions in patients with peripartum mental disorders

**DOI:** 10.1192/j.eurpsy.2024.354

**Published:** 2024-08-27

**Authors:** A. Bassi de Toni, G. Culicchia, A. Del Casale, M. Tinè, A. V. Vallerga, L. Cutillo, S. Bernardi, I. Bilotta, A. Fattorini, R. D’Alessio, D. De Felici, M. Pompili, G. Angeletti

**Affiliations:** ^1^Psychiatry Residency Training Program, Faculty of Medicine and Psychology; ^2^Department of Neurosciences, Mental Health and Sensory Organs, Faculty of Medicine and Psychology, Sant’Andrea Hospital, Sapienza University of Rome, Rome, Italy

## Abstract

**Introduction:**

Peripartum mental disorders (PPMD) are characterized by heterogeneous psychopathological symptoms related to specific personality traits, which are only taken into account by a few preventive and therapeutic strategies. Traumatic experiences during childhood could predispose to develop those disorders during adulthood, especially in more stressful conditions, such as pregnancy and postpartum.

**Objectives:**

Our study aims to evaluate the correlation between mother’s childhood trauma and the development of certain psychopathological dimensions during peripartum and which of these dimensions could be indicative of mother’s childhood trauma.

**Methods:**

The sample included 74 women, recruited from Sant’Andrea Hospital in Rome between 2011 and 2022, diagnosed with a psychiatric disorder during peripartum, according to criteria of DSM-5. All recruited women were administered the Childhood Trauma Questionnaire – Short Form (CTQ-SF) and the Minnesota Multiphasic Personality Inventory-2 (MMPI-2). We performed a linear regression using the total CTQ score as a dependent variable and the MMPI-2 scale’s scores as independent variables.

**Results:**

The linear regression used showed two significant models, of which the most inclusive explained 60% of the variance (R2 = 0.597), resulting significant (F = 31.141; p < 0.001). This model showed that a greater expression of childhood traumatic aspects was associated with greater expression of Pa (paranoia) (t = 4.04; p < 0.001) and Ma (hypomania) (t = 3.873; p < 0.001) in the clinical scales of the MMPI-2, which were indicative of childhood trauma.

**Conclusions:**

Our study shows that paranoiac and hypomanic symptoms in PPMD, assumed by the MMPI-2 scale, are indicative of previous traumatic dimension. Thus, in the presence of a positive history of trauma, clinicians should pay attention especially to these aspects, in order to optimally set both pharmacological and psychotherapeutic treatment.

**Disclosure of Interest:**

None Declared

